# Assessing the Efficacy and Safety of an 11β-Hydroxysteroid Dehydrogenase Type 1 Inhibitor (AZD4017) in the Idiopathic Intracranial Hypertension Drug Trial, IIH:DT: Clinical Methods and Design for a Phase II Randomized Controlled Trial

**DOI:** 10.2196/resprot.7806

**Published:** 2017-09-18

**Authors:** Keira Annie Markey, Ryan Ottridge, James L Mitchell, Caroline Rick, Rebecca Woolley, Natalie Ives, Peter Nightingale, Alexandra J Sinclair

**Affiliations:** ^1^ Neurometabolism, Institute of Metabolism and Systems Research College of Medical and Dental Sciences University of Birmingham Birmingham United Kingdom; ^2^ Centre for Endocrinology, Diabetes and Metabolism Birmingham Health Partners University of Birmingham Birmingham United Kingdom; ^3^ Birmingham Clinical Trials Unit University of Birmingham Birmingham United Kingdom; ^4^ Institute of Translational Medicine Queen Elizabeth Hospital Birmingham United Kingdom

**Keywords:** 11beta-HSD1, randomised controlled trial, clinical protocol, idiopathic intracranial hypertension, clinical trials, Phase II

## Abstract

**Background:**

Idiopathic intracranial hypertension (IIH) is a condition with few effective management options. So far, there have been no randomized controlled trials evaluating new treatments in IIH.

**Objectives:**

The purpose of this paper is to outline the trial design for the Idiopathic Intracranial Hypertension Drug Trial (IIH:DT), assessing an innovative medical treatment in IIH and the rationale for the chosen trial methodology.

**Methods:**

IIH:DT is a phase II double-blind randomized placebo-controlled trial recruiting 30 female participants with active IIH (intracranial pressure >25cm H_2_ O and papilledema). Participants are randomized in a 1:1 ratio to 12 weeks of either AZD4017, an 11β-hydroxysteroid dehydrogenase type 1 inhibitor, or a matching placebo. They receive either 400 mg of AZD4017 or placebo twice daily. Participants are followed up at Weeks 1, 2, 3, 4, 6, 8, 10, 12, and 16 postrandomization. The primary outcome is to examine the effect of AZD4017 on intracranial pressure, measured by lumbar puncture, over 12 weeks. Secondary outcome measures include IIH symptoms, visual function, papilledema, headache measures, safety, and tolerability. Cerebrospinal fluid, serum, plasma, urine, and adipose tissue are also taken for exploratory outcomes.

**Results:**

All participants were recruited between April 2014 and August 2016.

**Conclusions:**

IIH:DT is the first phase II double-blind randomized placebo-controlled trial assessing the efficacy and safety of the novel pharmacological intervention, AZD4017, for the treatment of IIH.

**Trial Registration:**

Clinicaltrials.gov NCT02017444; https://clinicaltrials.gov/ct2/show/NCT02017444 (Archived by WebCite at http://www.webcitation.org/6tVHesN6s)

## Introduction

Idiopathic intracranial hypertension (IIH), also known as benign intracranial hypertension or pseudotumor cerebri, is a condition of unknown etiology characterized by elevated intracranial pressure (ICP) and papilledema. IIH typically affects young obese females of child-bearing age, causing disabling daily headaches and visual loss. Among this population, the incidence of IIH is 12-20 per 100,000 [1,2] (0.5-2 per 100,000 in the general population [3,4]). In line with the global obesity epidemic, the incidence of IIH is expected to rise and consequently contribute to significant morbidity in young obese females.

A 2015 Cochrane review identified two randomized controlled trials (RCTs) assessing the use of acetazolamide in IIH [5]. The review included the Idiopathic Intracranial Hypertension Treatment Trial (IIHTT), which demonstrated beneficial effects of acetazolamide in IIH patients with mild visual loss [6], and a UK pilot study of acetazolamide [7]. Previous studies have highlighted elevated attrition rates [5] possibly due to drug side effects, which include paresthesia and gastrointestinal symptoms. The Cochrane review concluded that there was insufficient evidence to support the use of acetazolamide and that further well-designed RCTs are required [5].

For progressive or acute deterioration of vision in IIH, surgical techniques such as cerebrospinal fluid (CSF) shunting, optic nerve sheath fenestration, or venous sinus stenting have been used to prevent blindness. However, there is limited evidence to support these surgical interventions.

A 2010 prospective cohort study evaluated the effect of a low-calorie diet to promote weight loss in the treatment of IIH. The resulting dietary weight loss led to improvements in ICP, headaches, and vision [8]. Although weight loss is generally advised, the management of IIH varies considerably owing to a lack of supporting evidence [9,10]. Meaningful and sustained weight loss is also difficult to achieve. Consequently, alternative approaches are needed.

In the first phase II RCT in IIH ever conducted, the Idiopathic Intracranial Hypertension Drug Trial (IIH:DT) will assess whether the 11β-hydroxysteroid dehydrogenase type 1 (11β-HSD1) inhibitor AZD4017 is a safe and effective treatment for IIH.

### Scientific Background

The intracellular enzyme 11β-HSD1 converts inactive cortisone to active cortisol, amplifying local glucocorticoid availability independent of systemic circulating cortisol. AZD4017 is an oral selective, competitive inhibitor of 11β-HSD1 originally developed as a potential treatment for diabetes mellitus type 2, obesity, and metabolic syndrome.

Glucocorticoid metabolism has previously been characterized in IIH subjects before and after therapeutic weight reduction (loss of 15.7 kg [SD 8.0] of body weight) [8]. Global 11β-HSD1 activity decreased with weight loss as assessed by the urinary THF+allo-THF:THE ratio measured by gas chromatography/mass spectroscopy. Importantly, there was a relationship between the therapeutic reduction in ICP and the decrease in global 11β-HSD1 activity (*r*=.504, *P*=.03) [11].

These data not only highlight the potential link between 11β-HSD1 activity and ICP but offer the opportunity to test our hypothesis that specific inhibition of 11β-HSD1 will decrease ICP in IIH participants, opening an entirely novel therapeutic avenue.

Disordered CSF dynamics have been suspected of causing the raised ICP seen in IIH. The choroid plexus is the principle contributor to CSF production, which is ultimately driven by the sodium potassium ATPase (Na^+^K^+^ATPase) pump. Sodium transport creates an osmotic gradient driving water into the CSF [12]. Increasing the level of fluid within a closed system in turn increases ICP.

Aqueous humor produced by the ocular ciliary epithelium in the eye occurs by a mechanism analogous to CSF secretion in the choroid plexus, an embryologically related tissue [13]. A nonselective 11β-HSD inhibitor, carbenoxolone, was assessed in lowering ocular hypertension over 4 days of treatment, demonstrating a 10% (mean pressure: baseline 22.7, treatment 20.5, placebo 21.6; *P˂*.001) reduction in intraocular pressure compared to placebo. Inhibition of intracellular cortisol conversion within the ocular ciliary epithelium led to a decrease in sodium transport and a reduction in the osmotic gradient. As a consequence, water movement into the aqueous humor also decreased [14]. A similar process may occur at the choroid plexus with CSF secretion.

### Safety Considerations

AZD4017 is a fully reversible, competitive inhibitor of 11β-HSD1. It has been tested in two animal models: rats and nonhuman primates. The drug was found to be an effective inhibitor of 11β-HSD1 in human and nonhuman primates but a poor inhibitor in other animals [15]. AZD4017 was also tested in five phase I and II human clinical trials assessing the drug in healthy males, obesity, diabetes mellitus type 2, and raised intraocular pressure. The following safety considerations were found.

#### Liver

In animal models, reversible hypertrophy of the liver was observed in rats, which was adaptive rather than degenerative. However, nothing was noted in the cynomolgus monkey. Elevated transaminases, without concomitant rise in bilirubin, were observed in a few human subjects treated with AZD4017 in the multiple-ascending dose study. The findings were reversible on drug discontinuation, and no subjects were clinically symptomatic [15].

We hypothesize that specific inhibition of 11β-HSD1 will decrease ICP and consequently improve the symptoms and signs of IIH, providing a new pharmacological treatment for IIH.

#### Hypothalamic-Pituitary Axis

There were adrenal changes in the animal models, but these were considered adaptive rather than adverse. In the clinical studies, increases in adrenocorticotropin and dehydroepiandosterone- sulphate were seen, indicating an activation of the hypothalamic, pituitary, adrenal (HPA) axis. However, serum cortisol and testosterone levels were unchanged. There was no sign of adrenal insufficiency detected in humans [15].

#### Thyroid

Adaptive thyroid changes were seen in rats. In humans, there was no difference in thyroid hormones between AZD4017 and placebo-treated subjects. Despite this, thyroid function will be monitored owing to the preclinical results [15].

### Trial Objectives

The purpose of this paper is to outline the design considerations for IIH:DT. The aims of the trial are to assess the efficacy, safety, and tolerability of the selective 11β-HSD1 inhibitor AZD4017 for the treatment of IIH.

## Methods

### Trial Design

**Figure 1:**
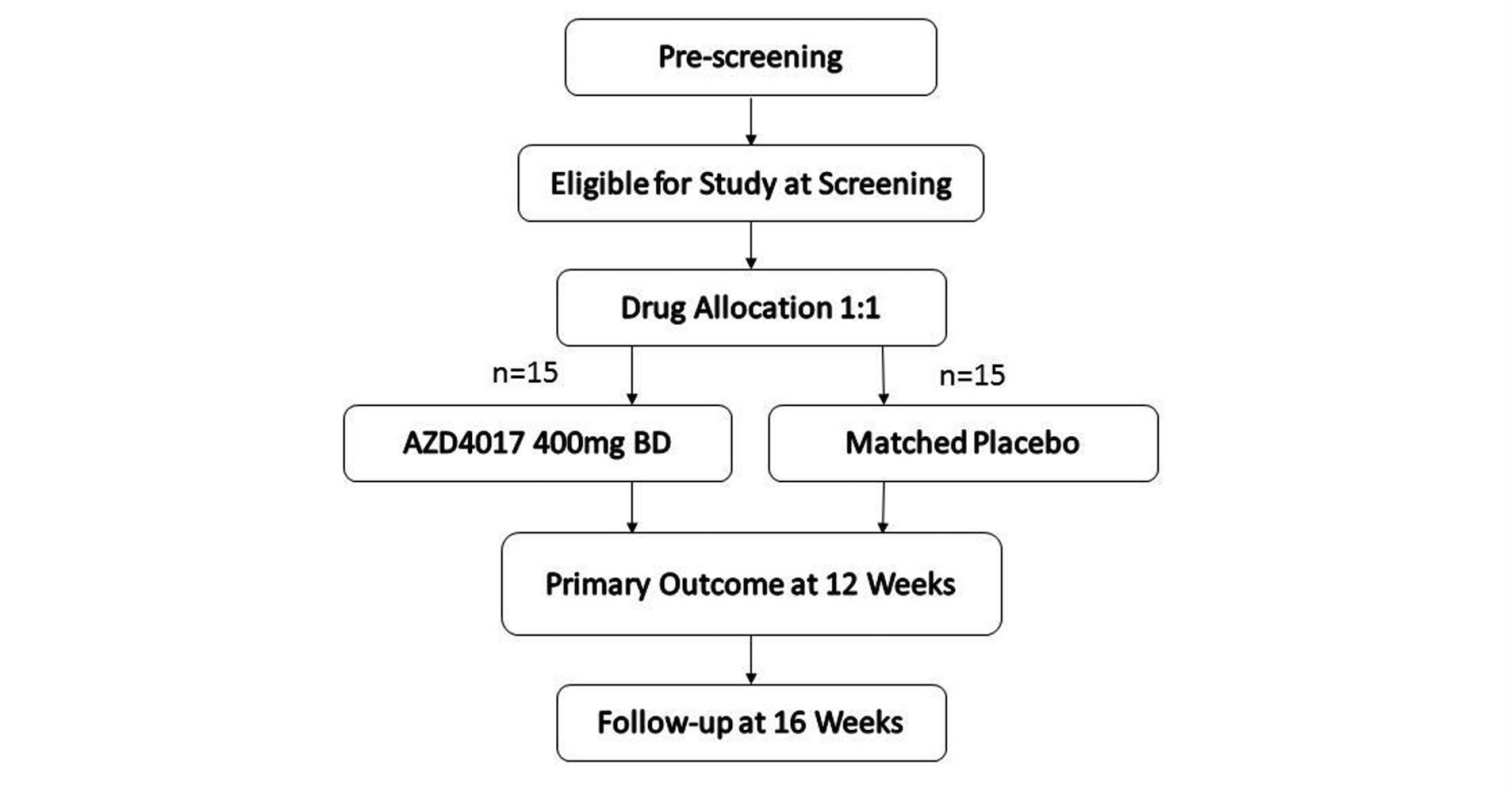
IIH:DT trial design.

IIH:DT is a multicenter phase II double-blind randomized, placebo-controlled trial in the United Kingdom comparing 12 weeks of treatment with 400 mg AZD4017 twice daily with a matching placebo in 30 female participants with active IIH (intracranial pressure [ICP] >25cm H_2_ O and papilledema). Participants are followed up for 16 weeks. The primary outcome examines the effect of AZD4017 on ICP, measured by lumbar puncture (LP), over 12 weeks (see Figure 1). An initial prescreening includes papilledema assessment and a blood test. Potential participants are invited to a screening 7-30 days later where an LP is performed. If eligible, random allocation to either AZD4017 or placebo occurs. Following 12 weeks of treatment, a repeat LP is performed. A final follow-up visit is then attended at Week 16.

### Recruitment

The aim of the trial was to recruit 30 participants (see sample size below) from four National Health Service (NHS) trusts across the United Kingdom between April 2014 and August 2016. Patient lists are screened before ophthalmology and research clinics, and those meeting the basic eligibility criteria approached at their appointment to determine their interest in trial participation. Consent is taken from willing patients to undertake prescreening. Prescreening consists of slit lamp examination for papilledema (Frisen grading ≥1) and a blood test to determine initial eligibility (see Table 1). If they continue to meet the eligibility criteria, they are asked to complete a 7-day headache diary (see Multimedia Appendix 1) and provide a 24-hour urine sample for glucocorticoid metabolite analysis. Participants are asked to return for a screening visit at least 7 days after their prescreening visit (see Trial Visits for further information).

### Eligibility

Patients are recruited based on the presence of active IIH, either newly diagnosed or a long-standing diagnosis, with any degree of visual loss and fulfillment of the trial inclusion and exclusion criteria (Table 1; [16]). As IIH predominantly affects obese females of child-bearing age, this will be the focus of the trial. Males and children tend to present differently with atypical features and may have an alternative pathogenesis [17] and so are excluded.

During the trial, the HPA hormones are monitored. We have excluded those on glucocorticoid and other hormonal medications as this may confound monitoring of the HPA axis. Those on mild topical steroid preparations or on inhaled steroids for asthma have been included as the systemic levels are low. The inclusion of IIH patients on acetazolamide and other diuretics is permitted if the participant is on a stable dose.

**Table 1:** Inclusion and exclusion criteria for IIH:DT.

Criteria	Description
Inclusion	Provision of informed consent prior to any study specific procedures
	Female patients 18-55 years
	Diagnosis of IIH by the Modified Dandy criteria [16] with active disease (papilledema [Frisen grade ≥1] and significantly raised ICP>25cm H_2_ O) and normal brain imaging during previous routine diagnostic work-up (evaluated by either magnetic resonance venography or computed tomography with venography)
	Participants must be willing to use one form of highly effective nonhormonal contraception. Participants must agree to undergo a urine pregnancy test at screening and at monthly intervals until the final follow-up visit 4 weeks after discontinuation of study treatment.
	Participants are able to continue other medications to treat their IIH (eg, acetazolamide, diuretics) but this dose should remain fixed throughout the study. Acetazolamide may be taken but the participant must be on a stable dose.
	Must be able to understand the consent form and comply with study requirements
Exclusion	Optic nerve sheath fenestration (as distortion of the optic nerve may prevent accurate assessment of their disease state). Participants who have had previous failed CSF shunting will be eligible for enrollment if they fulfill all other enrollment criteria.
	Abnormal neurological examination (aside from papilledema and consequent visual loss or VI nerve palsy)
	Unable to perform a visual field reliably
	Positive urine dipstick pregnancy test or planning to conceive in the 4 study months.
	Have estimated Glomerular Filtration Rate calculated by Modification of Diet in Renal Disease equation of <60ml/min/1.73m^2^
	Have any endocrine disorder, eg, thyroid dysfunction. Those with polycystic ovary syndrome will be included in the trial as there is a known association with IIH. Diabetes will not exclude participants.
	Suspicion of or known Gilbert’s disease
	Creatine kinase >2x upper limit of normal on 2 consecutive measurements
	Alanine transaminase and/or aspartate transaminase >2x upper limit of normal
	Alkaline phosphatase >upper limit of normal
	Bilirubin (total) >2x upper limit of normal
	Must not have donated blood within 2 months of screening and avoid further donation for 4 months following the study
	Participant is, at the time of signing the informed consent, a user of recreational or illicit drugs (including marijuana) or has had a recent history (within the last year) of drug or alcohol abuse or dependence, in the opinion of the investigator
	Pregnant or breastfeeding mother, unless willing to discontinue breastfeeding by baseline visit
	Have uncontrolled systemic hypertension (blood pressure >160 systolic on 3 successive measurements on the screening visit
	Are receiving systemic (including vaginal/rectal) glucocorticoid treatment at the time of the screening visit
	Are taking any hormone-based medication, including hormone contraceptives (but not including intrauterine system/hormonal coil), at the time of screening
	Are taking probenecid at the time of screening visit
	Have any screening laboratory abnormality that, in the investigator’s judgement, is considered to be clinically significant or any screening laboratory value that is outside the sponsor-specified ranges at screening; testing may be repeated once to see if the value returns to within the range, but any laboratory abnormality must be resolved prior to the baseline visit
	History of any clinically significant disease or disorder that, in the opinion of the investigator, may either put the subject at risk because of participation in the study or influence the results or the subject’s ability to participate in the study. Specifically, a diagnosis of any inflammatory disorder that might reasonably need treatment with glucocorticoids during the course of the study should be considered for exclusion.
	History or presence of significant gastrointestinal, hepatic^a^, or renal disease or any other condition known to interfere with absorption, distribution, metabolism, or excretion of drugs
	Any clinically significant illness, medical/surgical procedure, or trauma within 4 weeks of the first administration of the Investigational Medicinal Produce as judged by the investigator
	Have been involved in the planning and/or conduct of the study (applies to both AstraZeneca staff and/or staff at the study sites)
	Have participated in any other interventional studies within 1 month prior to the screening visit. Participation in the IIH national database or other observational studies will not prevent enrollment to this study.
	Previous randomization for treatment in this study

^a^A history of steatosis will not be considered an exclusion criterion.

### Ethics, Regulatory Approvals, and Dissemination

The trial will be conducted in accordance with the Research Governance Framework for Health and Social Care, the applicable UK Statutory Instruments (which include the Medicines for Human Use Clinical Trials 2004 and subsequent amendments), the Data Protection Act 1998, Human Tissue Act 2008, Human Tissue (Scotland) Act 2006, and Guidelines for Good Clinical Practice.

This trial will be carried out under a Clinical Trial Authorization (CTA) in accordance with the Medicines for Human Use Clinical Trials regulations. IIH:DT was approved by the National Research Ethics Committee York and Humber–Leeds West on November 19, 2013 (13/YH/0366), and received CTA for the use of investigational medicinal product on January 21, 2014. IIH:DT is sponsored by the University of Birmingham (RG 13-022). IIH:DT is registered on clinicaltrials.gov (NCT02017444) and the European Clinical Trials Database (EudraCT Number: 2013-003643-31.)

Results will be disseminated through internal reports, relevant conferences, peer-reviewed scientific journals, and online publications. No personally identifiable data will be published. Participants and general practitioners will be informed of results through a summary scientific report available through the trial website.

### Trial Drug and Placebo

The dose to be used in this trial is 400 mg taken as two 200 mg tablets, twice daily. Doses are taken 12 hours apart, typically around 7 a.m. and 7 p.m. each day. Participants take the trial medication for 12 weeks.

AZD4017 has been well tolerated in previous phase I and II studies. The largest single dose used has been 1200 mg for 9 days and the longest duration of use has been 28 days at 400 mg twice daily [15]. A 12-week treatment period is considered sufficient to see sustained inhibition of 11β-HSD1 by AZD4017 and potentially changes in ICP. This duration is longer than previous treatment with AZD4017; therefore, safety as well as drug efficacy is a key outcome.

AZD4017 is compared to placebo rather than a current treatment of IIH. During trial design and registration, there was no evidence supporting the use of any particular medical treatment for IIH [18]. While IIH:DT has been recruiting, new evidence emerged demonstrating the efficacy of acetazolamide in IIH patients with mild visual loss [6]. However, a recent systematic review suggested the evidence was inconclusive [5]. In addition, the IIH patient cohort included in this trial will include moderate to severe visual deficits. For these reasons, a placebo is still considered the optimal comparator for this trial.

Discontinuation of the trial drug can occur for a number of reasons: a clinically significant serious adverse event, deterioration in their IIH requiring CSF diversion surgery, severe noncompliance, hepatotoxicity, and muscle toxicity.

In the situation of very raised liver enzymes—alanine transaminase and/or aspartate transaminase >5 times the upper limit of normal (ULN) and/or bilirubin >2 times ULN and/or Hy’s Law criteria met—any time after randomization, the underlying cause for the liver enzyme elevation will be evaluated. If there is no improvement after 7 days or if the liver enzyme abnormality continues to worsen, depending on the clinical severity, then the treatment will be stopped. Subjects will also discontinue the trial drug if they fulfill all of Hy’s Law criteria for 2 weeks. The trial will be stopped completely if more than one Hy’s Law case persists for 2 weeks or more than three subjects (10%) meet the liver stopping criteria, unless another cause of liver enzyme abnormality is identified in these subjects.

### Randomization

Randomization has been performed by the contract manufacturing organization (Almac) supplying and coding the trial medication on behalf of AstraZeneca. Block randomization was used so that each block of trial numbers contains a random assignment of equal numbers of active and placebo treatment allocations.

Drug allocation takes place if the participant is confirmed to be eligible at the end of the screening visit and consents to enter the trial. The next available trial number is given to the participant in order. A drug pack, containing either AZD4017 or placebo, is identified by the designated trial number and will be provided to the patient on a monthly basis during the 12-week treatment period.

### Sample Size

We aimed to recruit 30 participants to allow for a sample size of 24 participants (12 per arm), with a 20% allowance for dropouts, which would allow 90% power (alpha=.05) to detect a difference of 14% in ICP (assuming a standard deviation of 10% for ICP).

### Outcomes

#### Primary Outcome

The primary outcome examines the effect of AZD4017 on ICP from baseline to 12 weeks postrandomization. ICP was chosen as the primary outcome to reflect the hypothesized action of AZD4017 inhibition of 11β-HSD1 with consequent reduction in CSF secretion and ICP.

#### Secondary Outcomes

Raised ICP underlies the symptoms and signs of IIH, thus alterations in ICP should result in clinical fluctuations. The following secondary outcomes are recorded:

- IIH symptoms (presence or absence of tinnitus, visual loss, diplopia, visual obscurations, and headache)
- IIH visual function in both eyes (measured by log of the minimum angle of resolution charts to assess visual acuity, automated perimetry [Humphrey 24-2 central threshold] to measure the visual field mean deviation and MARs charts to evaluate contrast sensitivity)
- papilledema is evaluated using spectral domain optical coherence tomography (Spectralis, Heidelberg Engineering) and fundal photographs with Frisen classification (by masked neuro-ophthalmologists) to grade the images
- headache-associated disability through the headache impact test-6 score (HIT 6), headache severity, frequency, and the use of analgesia (days/weeks)
- anthropological measures (blood pressure, body mass index, waist/hip ratio)
- safety and tolerability profile of AZD4017 through adverse event reporting and safety bloods (see Table 2)

#### Exploratory Outcomes

Exploratory studies evaluate the ability of AZD4017 to inhibit 11β-HSD1 in IIH patients through evaluation of:

- hepatic 11β-HSD1 activity by prednisone administration to measure prednisolone levels over 4 hours
- global 11β-HSD1 activity through urinary glucocorticoid metabolites
- 11β-HSD1 activity in subcutaneous adipose tissue following cortisone incubation ex vivo
- plasma and CSF drug levels and pharmacokinetic analysis
- cortisone and cortisol in serum and CSF

In addition, dual-energy x-ray absorptiometry (DXA) scanning is used to assess body habitus.

### Trial Visits

#### Screening

**Figure 2:**
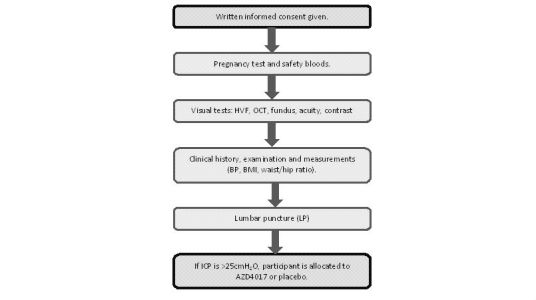
Overview of the screening visit.

A screening visit is attended between 7 and 30 days after successful prescreening. Participants are then assessed over 1 day or a split visit (see Figure 2). Following informed consent, a general history and examination as well as a neurological assessment is performed. A urinary pregnancy test and blood samples are taken followed by visual tests, DXA scanning, an LP, and fat biopsy. If the participant is eligible following screening, the data collected will be used as baseline data.

**Table 2:** A timeline of study visits and the outcome tests performed at each visit. Safety bloods include renal function (urea, creatinine, and electrolytes), liver function (aspartate transaminase, alanine transferase, bilirubin, albumin, alkaline phosphatase, gamma-glutamyl transferase), thyroid function (thyroid stimulating hormone free thyroxine), and creatine kinase.

Outcome		Measure	Weeks									
			0	1	2	3	4	6	8	10	12	16
**Primary outcome**												
	ICP	Lumbar puncture	X								X	
**Secondary outcomes**												
	Anthropometric measures	Body mass index, blood pressure, waist/hip, body composition	X								X	X
	IIH symptoms	Pulsatile tinnitus, visual loss, diplopia, visual obscurations	X								X	X
	Visual function	Visual acuity, contrast sensitivity	X								X	X
		Humphrey visual field (24-2)	X								X	X
	Papilledema	Optical coherence tomography	X								X	X
		Retinal photographs	X								X	X
	Headache	HIT-6, headache index, analgesia use	X								X	X
	Medical assessment	History, +/- examination, compliance	X	X	X	X	X	X	X	X	X	X
		Safety bloods	X	X			X		X		X	X
		Pregnancy test	X	X			X		X		X	X
**Exploratory outcomes**												
	Global 11β-HSD1 activity	24-hour urine	X	X			X		X		X	X
	Hepatic 11β-HSD1 activity	Prednisone to prednisolone measurements in serum	X								X	
	Adipose 11β-HSD1 activity	Subcutaneous adipose biopsy	X								X	
	Body habitus	DXA	X								X	
	Pharmacokinetic analysis	Plasma and CSF	X	X							X	

#### Subsequent Visits

The treatment period is 12 weeks. Participants will be assessed at 1, 2, 3, 4, 6, 8, 10, 12, and 16 weeks (see Table 2). All visits will determine adverse events and treatment compliance. Visits 1, 4, 8, 12, and 16 will require safety and hormone bloods as well as a urinary pregnancy test. The 12-week visit will repeat the screening visit. Following 4 weeks off the trial drug, patients will be assessed at 16 weeks for visual tests, headaches, bloods, IIH symptoms, and adverse events. The follow-up visit at Week 16 is deemed sufficient time for the effects of the trial drug to have dissipated. Any relapses in IIH-related symptoms are then determined.

### Statistical Methods

The primary analysis of efficacy is based on the full analysis set, as defined by ICH guidance document *E9: Statistical Principles for Clinical Trials*. This analysis adheres as closely as possible to the intention-to-treat ideal and is based on data from all randomized participants with participants analyzed in the treatment group to which they were randomized.

### Primary and Secondary Outcome Analyses

The primary comparison groups are composed of those randomized to AZD4017 versus placebo. For all major outcome measures, summary statistics and differences between groups (eg, mean differences) will be presented with 95% confidence intervals and *P* values from two-sided tests. No adjustment for multiple comparisons will be made.

The primary outcome examines the effect of AZD4017 on ICP from baseline to 12 weeks postrandomization. Data will be reported with means and standard deviations or medians and ranges for nonparametric data. The ICP at 12 weeks for the two trial arms will be compared using a linear regression model with baseline ICP included as a covariate in the model.

Secondary outcome measures are analyzed at 12 and 16 weeks. The majority of secondary outcomes are continuous data items, which will be analyzed as per the primary outcome. For the visual function data that are collected in both eyes, it is expected that the participant’s data will be correlated, so the primary analyses will use data from both eyes and will be analyzed using a linear mixed model, with participants included as a random effect. The visual function data for each eye will also be analyzed separately as per the primary outcome, but this will be a secondary analysis. The IIH symptom data is binary and will be analyzed using log-binomial models with baseline symptom included in the model as a covariate.

### Study Withdrawal

Participants are free to withdraw from the study at any time. Such participants will always be asked about the reason(s) and the presence of any adverse events. If the participant is withdrawn from the trial by the investigator for any reason at any visit during the study, assessments for the early discontinuation visit will be completed during that visit, and the withdrawn participant asked to return for Visit 12 between 83 and 97 days, inclusive.

If the participant needs to be withdrawn from the active treatment phase between trial visits for safety reasons, trial medication will be stopped immediately and the participant will be instructed to attend the next arranged clinic visit, which will be treated as an early discontinuation visit. If it is considered appropriate by the investigator, more frequent or additional follow-up visits can be scheduled (if willing, the participant will be asked to attend the 12-week final trial visit). Withdrawn participants will not be replaced.

### Monitoring

#### Adverse Event Reporting

All serious adverse events (SAEs) must be reported, whether or not considered causally related to the trial drug or to study procedures. A clinician must assess the severity, causality, and expectedness of the SAE. If the event is considered to be related to the study drug, then the SAE will be classed as a serious adverse reaction (SAR). If the SAR is listed in the safety information of AZD4017, then it is listed as expected and included in the ongoing safety review of the study. If it is not an expected SAR for AZD4017, then it is listed as a suspected unexpected serious adverse reaction (SUSAR). The trial team will then be contacted to unblind the subject’s medication allocation. A fatal or life-threatening SUSAR will be reported to the Medicines and Healthcare Products Regulatory Agency and the research ethics committee within 7 days.

#### Unblinding

Investigators and subjects are unaware whether the product is AZD4017 or placebo (double-blinded); this information is held by local pharmacies in the form of scratchcards detailing the trial number and a scratch-off panel. Unblinding can take place in the case of a SUSAR and in medical emergencies, when knowing the treatment will change the management of the patient.

#### Monitoring Committees

A trial steering committee made up of independent members (including the chair) and trial team members provides oversight of the trial. An independent data monitoring committee (DMC) monitors unblinded efficacy and safety reports. If there are any major safety concerns, the DMC can recommend early discontinuation of the trial.

## Results

The trial opened March 2014 with the first patient recruited April 2014. Additional sites were opened at the Walton Centre, Liverpool (January 2016), Southern General Hospital, Glasgow (March 2016), and Royal Hallamshire Hospital, Sheffield (April 2016) to facilitate recruitment. The DMC and trial steering committee meet regularly to monitor the course of the IIH:DT. The trial results are expected in September 2017.

## Discussion

### Principal Considerations

Recruitment to IIH:DT was completed in August 2016. Results from the trial are still awaited. The two RCTs evaluating IIH treatments completed thus far have investigated an established drug, acetazolamide. IIH:DT is the first to assess the efficacy of a new drug treatment, an 11β-HSD1 inhibitor (AZD4017), not tested in IIH before. More importantly, it aims to establish the safety profile of AZD4017 in IIH. Safety monitoring involves regular safety bloods and adverse event recording. These are performed in accordance with prior AZD4017 study results. This is the longest period of time the drug has been administered to humans. Positive safety results will provide important information for future trials using AZD4017.

### Conclusions

IIH is in need of new treatments. IIH:DT is the first phase II double-blinded randomized placebo-controlled trial in IIH. It assesses a novel therapeutic pathway through inhibition of 11β-HSD1 by the drug AZD4017. In addition to the primary outcome of ICP, a range of secondary outcomes including visual function, headache, and papilledema will be assessed. IIH:DT aims to establish initial evidence of efficacy and safety that could lead to a future large phase III study using AZD4017 in IIH.

## Appendix

Multimedia Appendix 1 IIH:DT headache diary.

Multimedia Appendix 2 UK Medical Research Council (MRC) Reviewer comments.

## Abbreviations

BCTU: Birmingham Clinical Trials Unit
CSF: cerebrospinal fluid
CTA: clinical trial authorization
DMC: data monitoring committee
DXA: dual-energy x-ray absorptiometry
HIT-6: headache impact test 6
HPA: hypothalamic, pituitary, adrenal
11β-HSD1: 11beta-hydroxysteroid dehydrogenase type 1
ICP: intracranial pressure
IIH: idiopathic intracranial hypertension
IIH:DT: Idiopathic Intracranial Hypertension Drug Trial
IIHTT: Idiopathic Intracranial Hypertension Treatment Trial
LP: lumbar puncture
MRC: Medical Research Council
NHS: National Health Service
RCT: randomized controlled trial
SAE: serious adverse event
SAR: serious adverse reaction
SUSAR: suspected unexpected serious adverse reaction
