# Supplementary material for: Assessing the Efficacy and Safety of an 11β-Hydroxysteroid Dehydrogenase Type 1 Inhibitor (AZD4017) in the Idiopathic Intracranial Hypertension Drug Trial, IIH:DT: Clinical Methods and Design for a Phase II Randomized Controlled Trial
Source: JMIR Res Protoc. 2017 Sep 18;6(9):e181. doi: 10.2196/resprot.7806 (PMC5625129; doi:10.2196/resprot.7806)
Supplement: Multimedia Appendix 1 [file resprot_v6i9e181_app1.pdf]

[To be printed on pink card]

The impact of AZD4017 on Intracranial pressure and Symptoms of Idiopathic Intracranial Hypertension

IIH:DT Headache Diary

Patient's Name ____________________________________

Patient's Study Number ____________________________

Date and Visit _____________________________________

Contact: Monday–Friday <<insert local contact hours and telephone number>>

Any other time - **Emergency contact**

<<insert local switchboard>> (Switchboard)

Please ask for the Neurology on-call doctor

PLEASE BRING THIS DIARY WITH YOU TO THE VISITS INDICATED ON YOUR APPOINTMENT DIARY (YELLOW CARD).

[To be printed on pink card]

[illegible]

Please record EVERY DAY:

The **severity** of your headache from 0 (no headache) to 10 (worst pain ever experienced)

The **total duration** of your headaches in hours (**e.g.** 4 hours)

The **number** and **type** of **pain killers taken**.

If you have **not** taken any painkillers, please write **‘none’** in the painkillers section.

DAY	DATE<br>DD-MMM-YYYY	SEVERITY<br>(0-10)	DURATION<br>(hours)	PAINKILLERS<br>(Type and Number)
1			_____hours	___________________<br>___________________
2			_____hours	___________________<br>___________________
3			_____hours	___________________<br>___________________
4			_____hours	___________________<br>___________________
5			_____hours	___________________<br>___________________
6			_____hours	___________________<br>___________________
7			_____hours	___________________<br>___________________
