# Supplementary material for: Assessing the Efficacy and Safety of an 11β-Hydroxysteroid Dehydrogenase Type 1 Inhibitor (AZD4017) in the Idiopathic Intracranial Hypertension Drug Trial, IIH:DT: Clinical Methods and Design for a Phase II Randomized Controlled Trial
Source: JMIR Res Protoc. 2017 Sep 18;6(9):e181. doi: 10.2196/resprot.7806 (PMC5625129; doi:10.2196/resprot.7806)
Supplement: Multimedia Appendix 2 [file resprot_v6i9e181_app2.pdf]

Panel Assessment Template

IN CONFIDENCE

Medical Research Council	Astra Zeneca mechanisms of disease initiative (AZ)	4th October 2012
	File reference:	MR/K015184/1
	Minute:	Item 15

BACKGROUND INFORMATION

PRINCIPAL INVESTIGATOR:	Dr Alexandra Sinclair
DEPARTMENT/LOCATION:	University of Birmingham
TITLE OF GRANT/NAME OF UNIT:	Assessing the therapeutic efficacy of an 11beta-hydroxysteroid dehydrogenase type 1 inhibitor (AZD4017) in idiopathic intracranial hypertension (IIH).
FORM OF SUPPORT:	Research Grant
START DATE & DURATION:	01/06/2013 & 36 months
NAME OF ESS: DATE OF UNIT/ESS VISIT, IF ANY:	

PANEL SCORE:	7.0	TOTAL MRC CONTRIBUTION REQUESTED(£K, INDEXED):	£374,946
		DECISION:	Awarded in principle

NOTE: In the case of direct support (e.g. for MRC units) costs represent changes to the current level of funding.

ABSTRACT

Idiopathic intracranial hypertension (IIH), is a common condition of unknown aetiology affecting the young obese female population (20 per 100,000). Faced with the global obesity epidemic, its incidence is expected to rise further. IIH is characterised by elevated intracranial pressure (ICP) which manifests as disabling headaches and severe visual loss in up to 25% of patients. An evidence base for treatment has not been established and there are currently no effective and tolerable pharamcotherapeutic options. Based upon our in-vitro observations, we have hypothesised that increased local glucocorticoid generation through the activity of 11beta-hydroxysteroid dehydrogenase type 1 (11beta-HSD1) within the choroid plexus that generates cerebrospinal fluid (excess production leading to raised ICP) is crucial to the pathogenesis of IIH. This mechanism is analogous to that occurring in the ocular ciliary body which produces aqueous humor where 11beta-HSD1 inhibitors have been shown to lower intraocular pressure. We have recently published a seminal study in patients with IIH and demonstrated that weight loss significantly reduced 11beta-HSD1 activity, ICP and dramatically improved patient symptoms; the observed reduction in ICP correlated directly with the reduction in ICP.

In collaboration with our industrial partner, AstraZeneca (AZ), we will conduct a double blind, placebo controlled randomised phase II study to assess the tolerability, safety and efficacy of the selective 11beta-HSD1 inhibitor, AZD4017, in the treatment of IIH. The study will be conducted at University Hospitals Birmingham NHS Foundation Trust, the Midlands Eye Centre and the Wellcome Trust Clinical Research Facility. In total, 24 patients with active chronic IIH (raised ICP and papilloedema) will be randomised to AZD4017 or placebo tablets (1:1).The primary outcome measure, intracranial pressure, will be evaluated by lumbar puncture at 12 weeks compared to baseline.

PANEL ASSESSMENT

Assessment of the Proposed Research

This was a very good application from one of the leading groups in the field.

Whilst discussing the proposal the following points were noted:

- The proposal addressed an important area of clinical unmet need.
- Whilst the proposal was well written there appeared to be a slight lack of mechanistic insight.
- Members agreed that the proposal lacked sufficient justification for a full time technician and agreed that this post should be reduced to a half-time position.
- It was agreed that the applicants were missing an opportunity in not studying whether the drug crossed the blood brain barrier and investigating what the possible effects of the drug were on shunt patients. As such, Members asked that the applicant include studies that measured drug levels in the CSF and studies that assessed the effects of the drug on shunt patients.
- Members raised concerns regarding the statistically analyses put forward and it would be important to carefully consider sample size calculations to ensure whether more patients would need to be recruited. However, Members acknowledged that given that this was not the most common of conditions patient recruitment may be challenging.

In conclusion, the Panel agreed that this was a strong application and agreed to award it subject to the submission of revised costings to take into account the comments outlined above.

Level of Support Recommended

Award at revised level following submission of:

- Revised costing as outlined above
- Collaboration agreement
